# Supplementary figures and images for: American public opinion on artificial intelligence in healthcare
Source: PLoS One. 2023 Nov 9;18(11):e0294028. doi: 10.1371/journal.pone.0294028 (PMC10635466; doi:10.1371/journal.pone.0294028)

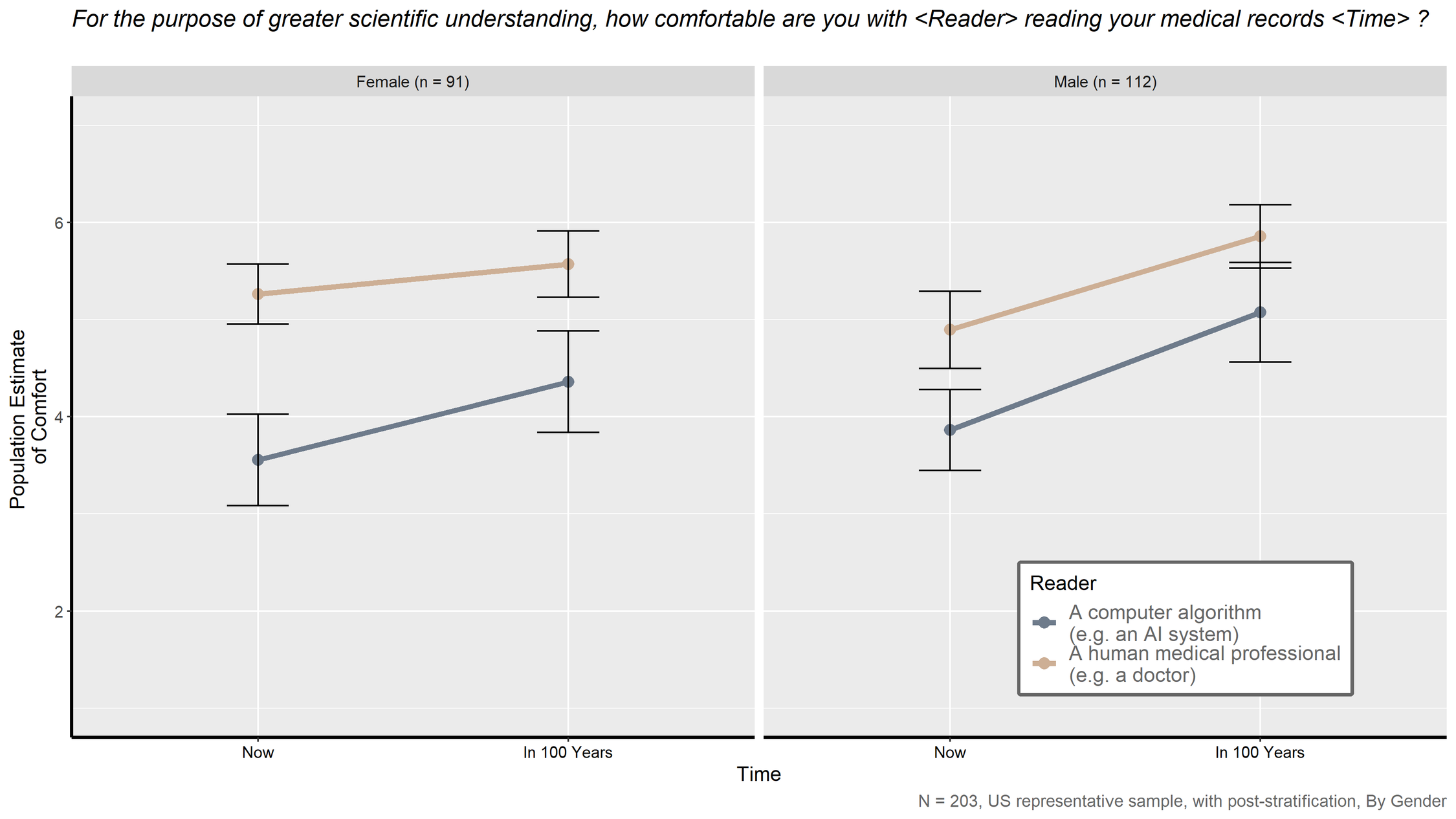

Supplement: S1 Fig — A computer algorithm (e.g. an AI system). A human medical professional (e.g. a doctor). By gender. (TIF) [file pone.0294028.s001.tif]

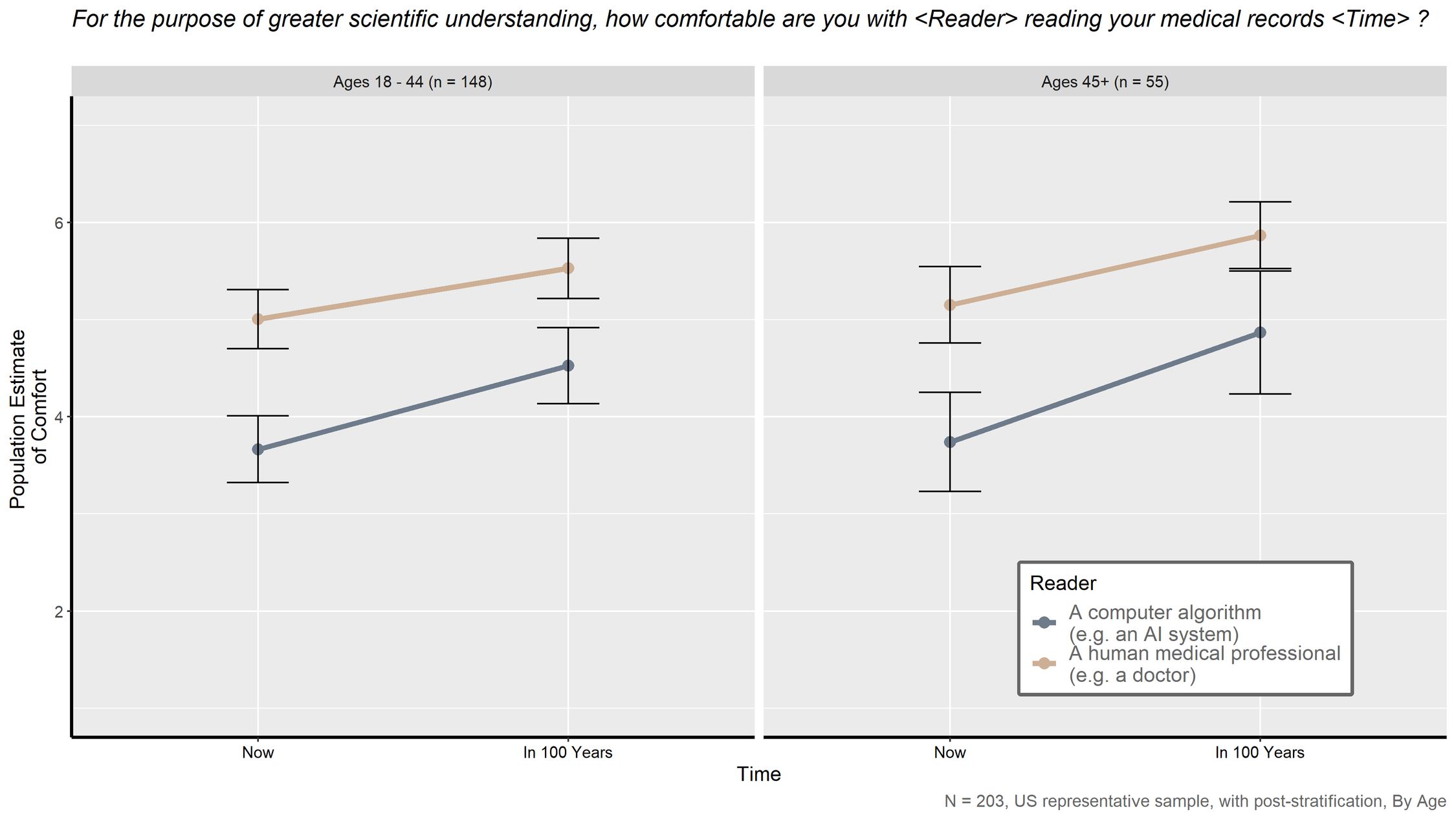

Supplement: S2 Fig — A computer algorithm (e.g. an AI system). A human medical professional (e.g. a doctor). By age. (TIF) [file pone.0294028.s002.tif]
