# Supplementary material for: American public opinion on artificial intelligence in healthcare
Source: PLoS One. 2023 Nov 9;18(11):e0294028. doi: 10.1371/journal.pone.0294028 (PMC10635466; doi:10.1371/journal.pone.0294028)
Supplement: Attachment — Submitted filename: PLOSOne_Response_Letter_MedAI.docx [file pone.0294028.s003.docx]

Jessica Rojahn
Stony Brook University
100 Nicolls Rd, Stony Brook, NY 11794

March 24, 2023

Dear Editor,

Thank you very much for the reviews on the previous version of the manuscript. We are submitting today a revised version and this response letter addressing all the reviewer points and journal requirements.

There is one additional important point in our revision. A previously listed author (Skiena) would prefer to be not listed as an author. His contribution (as listed in the original author contributions) was "Funding, Administration and Resources." He feels that is not sufficient to be listed as an author of the manuscript, and requests that his authorship be removed. You may contact him directly to confirm.

Thank you for your time and attention.

Sincerely,

Jessica Rojahn

page 3 - Introduction section: please provide a more complete definition of artificial intelligence e provide an example to how it is applicable to different field of medicine ( Lavorgna L, Bonavita S. Artificial intelligence will change MS care within the next 10 years: Commentary. Mult Scler. 2022 Dec;28(14):2175-2176. doi: 10.1177/13524585221133537. PMID: 36408580.)

Please find on page 3 an expanded definition of artificial intelligence. Thank you for the suggested manuscript. Unfortunately, our library was not able to obtain it, and we did not receive it after an email and ResearchGate request to the corresponding author. We have included an example of use of AI in dermatology in the revised manuscript.

1. Please ensure that your manuscript meets PLOS ONE's style requirements, including those for file naming.

We have ensured the manuscript meets PLOS ONE's style requirements.

2. Please provide additional details regarding participant consent. In the ethics statement in the Methods and online submission information, please ensure that you have specified (1) whether consent was informed and (2) what type you obtained (for instance, written or verbal, and if verbal, how it was documented and witnessed). If your study included minors, state whether you obtained consent from parents or guardians. If the need for consent was waived by the ethics committee, please include this information.

The study did not involve minors.

We have added the following text to the Method section: Respondents consented by accepting and completing the online survey. They could opt out at any time without penalty. The identity of the respondents cannot readily be ascertained, directly or through identifiers linked to the subjects. The survey data collection qualifies as Exempt research under the criteria specified in Department of Health and Human Services code § 46.104(d)(2).

If you are reporting a retrospective study of medical records or archived samples, please ensure that you have discussed whether all data were fully anonymized before you accessed them and/or whether the IRB or ethics committee waived the requirement for informed consent. If patients provided informed written consent to have data from their medical records used in research, please include this information.

There were no medical records or archived samples.

3. Please include your full ethics statement in the ‘Methods’ section of your manuscript file. In your statement, please include the full name of the IRB or ethics committee who approved or waived your study, as well as whether or not you obtained informed written or verbal consent. If consent was waived for your study, please include this information in your statement as well.

We have included an Ethics Statement subsection within Methods.

4. Please review your reference list to ensure that it is complete and correct. If you have cited papers that have been retracted, please include the rationale for doing so in the manuscript text, or remove these references and replace them with relevant current references. Any changes to the reference list should be mentioned in the rebuttal letter that accompanies your revised manuscript. If you need to cite a retracted article, indicate the article’s retracted status in the References list and also include a citation and full reference for the retraction notice.

We have reviewed the reference list to ensure that it is complete and correct
