# Supplementary material for: American public opinion on artificial intelligence in healthcare
Source: PLoS One. 2023 Nov 9;18(11):e0294028. doi: 10.1371/journal.pone.0294028 (PMC10635466; doi:10.1371/journal.pone.0294028)
Supplement: Attachment — Submitted filename: PLOSOne_Response_Letter_MedAI_09032023.docx [file pone.0294028.s004.docx]

Jessica Rojahn
Stony Brook University
100 Nicolls Rd, Stony Brook, NY 11794

September 3, 2023

Dr. Mahmoud,

Thank you very much for the reviews and request to revise. We are submitting today a revised version and this response letter addressing all the reviewer points and journal requirements.

Thank you for your time and attention.

Sincerely,

Jessica Rojahn

Please review your reference list to ensure that it is complete and correct. Any changes to the reference list should be mentioned in the rebuttal letter that accompanies your revised manuscript.

We have reviewed the reference list to ensure that it is complete and correct.

We removed a duplicate reference from the reference list and updated the list order.

There are a couple of English errors:

Abstract, line 3:

high expectations for the future of medical AI does exist ..--> .... do exist.

We have corrected this error.

Page 13 line 2:

previous data does not exist --> .. do not exist

We have corrected this error.

Page 13 line 8:

interpreted by the subjects of those decisions --> the word subject is confusing, it seem to indicate the people who made the decisions instead of the people for which the AI made the decisions.

We have reworded this sentence to make it more clear.

Overall, the results of the questionnaire look somehow trivial and expected. The people who answered the questions are unlikely to have been exposed to a decision on their health made by AI, so with no first hand experience they produced obvious answers.

We strongly disagree with these unfounded assertions. The reviewer provides no evidence the “results of the questionnaire” are either trivial or expected. The reviewer does not mention any specific result, nor any reason the variable responses to the multiple items were “obvious.”

We have made no revisions in response to these comments, because it is impossible to retroactively surprise the reviewer.

It would have been useful to ask the subjects if they had any personal experience with an AI application used for health. A more rich set of questions, perhaps including some short videos of doctors and of AI systems, would likely have elicited different answers. The authors could have asked a few additional questions to be able to refine their analysis of the answers and get more insights on the opinions of the people who took the survey.

The reviewer has a different instrument in mind. It is not clear to us that short videos of doctors and short videos of AI systems would add any value to the current instrument. One can always imagine additional items on any survey. Here we were constrained to exactly 10 items due to the survey platform.

The assertion that videos “would likely have elicited different answers” is an empirically testable proposition. It is outside the scope of the current work, and the authors doubt the reviewer’s assertion.

We have made no revisions in response to these comments.

What is interesting is that despite the fact people think of AI as being less empathetic than a person, some large language models are being used to train the doctors to be more empathetic when they talk with their patients.

This is a potentially interesting aside. We would be interested in a reference to whatever work the reviewer is thinking of. However, none of the current results address perceptions of empathy.

We have made no revisions in response to these comments.

The paper does not say when the survey was conducted, which is important since AI advances are reported almost daily by the press, and that can change the opinions of the general public.

We thank the reviewer for pointing out this oversight. The data was collected in March 2021. We have added this information to the Methods section.

Also, the number of subjects is relatively small to make inferences about the US population.

When statistics are properly implemented, the phrase “the number of subjects is relatively small to make inferences” is devoid of meaning. We have included confidence intervals on every estimate and a p-value for each statistical test. Of course, the common pitfalls of sample-to-population inference apply to the current results as they do to every published quantitative result.

A scale to measure attitudes toward AI is described in: Schepman, A., & Rodway, P. (2020). Initial validation of the general attitudes towards Artificial Intelligence Scale. *Computers in human behavior reports*, *1*, 100014. This scale is published in a peer-reviewed journal and has been cited 101 times. The validation in Schepman & Rodway (2020) is based on 100 respondents. In the current results, we surveyed 203 respondents.

We have made no revisions in response to this comment.

Per the Editor’s request, we have submitted this response letter, a marked-up copy of the manuscript that highlights changes made, and an unmarked version of the revised paper without tracked changes.

We look forward to a decision from the editor following our revisions and this response.
